# Recent Advances in the Chemical Recycling of Polyurethane Foam from End-of-Life Vehicles: A Review

**DOI:** 10.3390/polym18182224

**Published:** 2026-09-12

**Authors:** Aleksi Modebadze, Vladimír Sedlařík

**Affiliations:** Centre of Polymer Systems, University Institute, Tomas Bata University in Zlín, Tř. T. Bati 5678, 760 01 Zlín, Czech Republic; sedlarik@utb.cz

**Keywords:** polyurethane foam, chemical recycling, glycolysis, acidolysis, end-of-life vehicle, circular economy

## Abstract

Flexible polyurethane (PU) foam is the main cushioning material in a modern vehicle interior, filling the seat cushions, headrests, headliners, and acoustic insulation, yet it is among the least recovered fractions of an end-of-life vehicle (ELV). The foam is a crosslinked thermoset and cannot be remelted; poor separation and sorting, compounded by contamination during dismantling and shredding, leave incineration or landfill as the most probable outcome. Two recovery routes are established: mechanical recycling, which reuses the foam in ground or rebonded form, and chemical recycling, which cleaves the urethane linkage to recover the polyol. Most recovery advances have been demonstrated on mattress and industrial scrap foam rather than on automotive foam itself, though studies on genuine automotive foam are beginning to emerge, and glycolysis and acidolysis stand out among the chemical routes as the most promising for this feedstock. This review therefore surveys recent advances in PU foam recycling with a focus on automotive foam and, because direct studies on this feedstock remain few, also assesses how far the wider recycling literature applies to it. Recovered-polyol quality, achievable blend ratios, industrial implementations, and the European regulatory framework are examined.

## 1. Introduction

**Figure 1:**
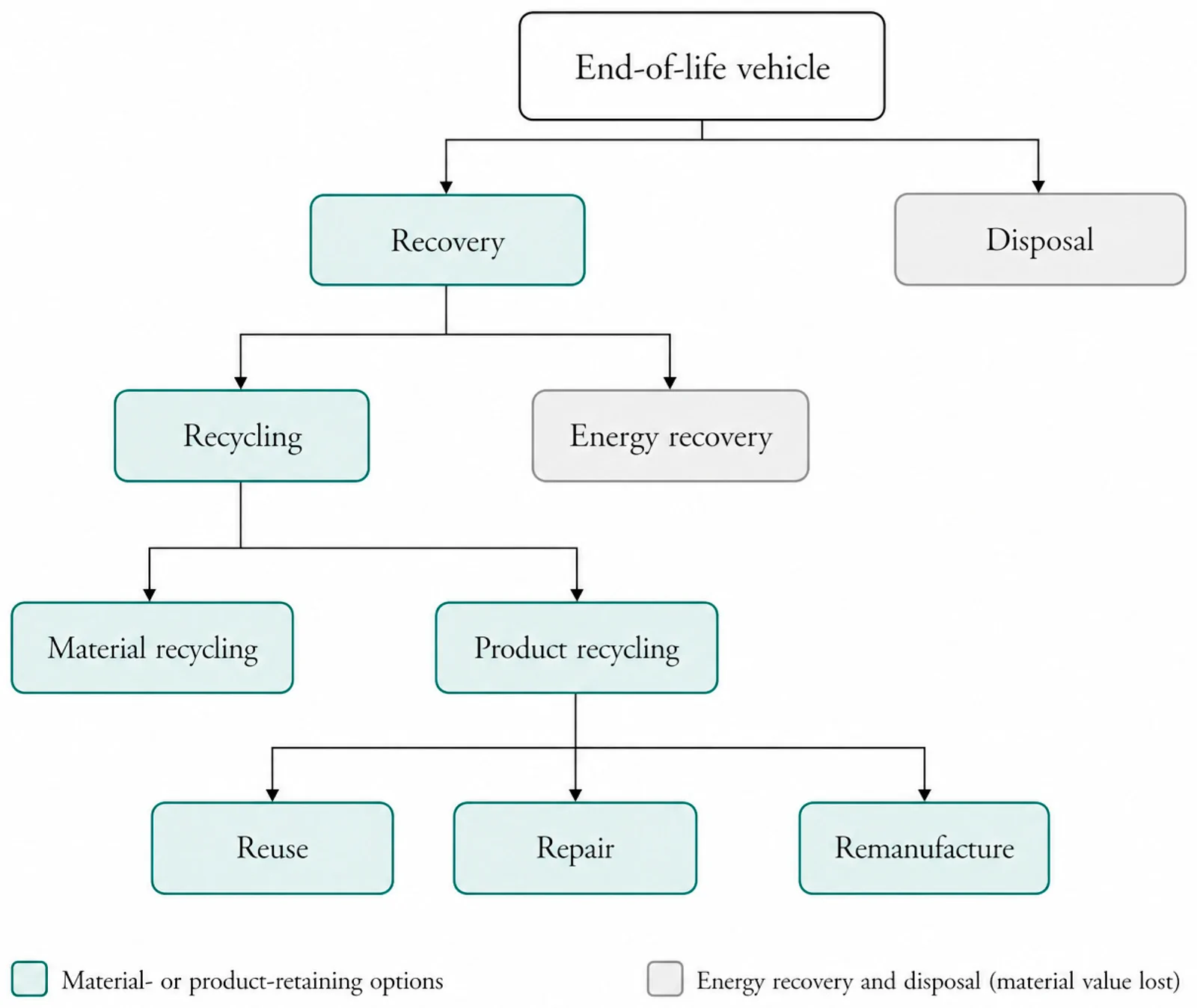
Waste treatment options for end-of-life vehicles, based on the classification presented in [8].

Polymers rank as the second most prevalent material class in a vehicle, making up between 5.8% and 10% of its composition, a share that has grown substantially to approach 160 kg in a typical passenger car [1,2]. Four polymer families dominate this fraction: polypropylene (PP), polyurethane (PU), polyamide (PA), and poly(vinyl chloride) (PVC) [2]. These same vehicles, once they reach the end of their service life and become what is termed an end-of-life vehicle (ELV) [3], join a waste stream of considerable scale: global generation was projected to approach 80 million units per year by 2020 [4], and more recently, 4.3 million vehicles were scrapped in the EU alone in 2023 [5], with the vehicle sector contributing about 5% of the world’s industrial waste [6]. Roughly three-quarters of a vehicle’s weight, mainly steel and aluminium, is readily recycled, but the polymer fraction is scattered through the structure and often bonded to other materials, which makes it far harder to recover [7]. The options for treating this waste form a hierarchy, shown in Figure 1, in which recovery is preferred to disposal and, within recovery, material recycling that returns usable feedstock ranks above energy recovery that retains only the calorific value [8]. The higher a material sits on this hierarchy, the more of its worth survives. This makes the polymer fraction central to the sector’s move away from disposal and toward a circular model. Regulation has pushed that change: European law has governed end-of-life vehicle treatment for more than two decades. Directive 2000/53/EC sets firm targets, requiring 85% of a vehicle’s weight to be recycled or reused and 95% to be recovered [9]. Because these targets are defined by weight, and metals make up close to three-quarters of a vehicle, they can be attained while much of the lighter polymer fraction stays outside material recycling; it has been noted that the current targets can be reached with almost no recycling of plastics [10]. That loophole is closing. In December 2025, the European Parliament and Council reached a tentative agreement on a new ELV Regulation to replace the Directive, and for the first time it will require manufacturers to build recycled plastic into new vehicles, some of it drawn from the vehicles themselves [11]. Supplying that recycled plastic means looking closely at each polymer a vehicle contains.

Among the polymers that could supply this feedstock, polyurethane occupies a particular position: it is one of the most widely used, and its dominant product form, foam, is one of the most difficult to recover. The chains of a polyurethane foam are joined into a covalently crosslinked network, which makes it a thermoset, and unlike the thermoplastics that constitute most of a vehicle’s plastic, it cannot be melted and reprocessed, so its recovery requires dedicated treatments [12]. Despite this constraint, its properties can be tuned, through the choice of raw materials and formulation, across a wide range from soft and flexible to hard and rigid [13], and this versatility has built a substantial market: the European polyurethane industry generates some €149 billion annually across furniture, mattresses and bedding, thermal and acoustic insulation, packaging, and vehicle interiors [14]. Mattresses show what this waste looks like once it accumulates unmanaged: discarded units already form one of the largest bulky-waste flows in the European Union, and most are landfilled or incinerated rather than recovered [15]. Because vehicle and mattress foam share this crosslinked structure, the mattress stream previews, at a larger and better-documented scale, the disposal problem the automotive sector is only beginning to confront, and recovering the foam represents both an environmental necessity and a source of the recycled feedstock vehicle manufacturers will shortly be required to find. Even the automotive thermoplastics that can, in principle, be remelted rarely achieve closed-loop recycling from end-of-life vehicles [16], and for foam the difficulty is compounded further by the contamination and additives the material carries [17]. The treatments available are divided broadly into two routes: mechanical routes reuse the foam in a physically altered, lower-value form, while chemical routes depolymerise the network to recover the constituent polyol. Of the two, chemical recovery offers the closest approach to a genuine closed loop and has attracted the greatest research attention [18]. Even so, the recovered polyol has generally replaced only part of the virgin material in reconstituted foam, and whether chemical recycling can deliver foam of comparable quality remains an open question [19].

These open questions trace back to what the underlying studies actually used as feedstock. Almost all of the chemical-recycling literature has been built on mattress foam and industrial scrap, and only a handful of studies, such as a recent industrial-scale glycolysis of post-consumer automotive foam, have worked with genuine car seat or headrest material; these remain isolated exceptions rather than an established body of work [20]. This intersection has not previously been examined as a whole. End-of-life vehicle management has been reviewed in its own right [4], and the chemical recycling of polyurethane has attracted a substantial review literature of its own, with glycolysis chief among the routes discussed, yet the two strands have not been brought together. Whether the polyol liberated from automotive foam is equally susceptible to established depolymerisation routes, or whether its contaminants hinder the reaction and downgrade the recovered precursor, is a question the existing literature leaves largely unaddressed. Because direct studies of automotive foam remain few, the present review responds to this gap by surveying the most recent and emerging advances in polyurethane foam recycling from end-of-life vehicles alongside the other feedstocks, chiefly mattress and industrial scrap, on which most of the field’s progress has been demonstrated, with particular attention to glycolysis and acidolysis, the two routes suggested by the literature as most transferable to automotive foam; hydrolysis and aminolysis are noted only briefly, since neither is yet developed enough to justify closer treatment. Relevant literature was identified through the Scopus, Web of Science, and Google Scholar databases, using combinations of the keywords “polyurethane foam”, “chemical recycling”, “glycolysis”, “acidolysis”, “end-of-life vehicle”, and “circular economy”, with priority given to publications from 2020 onward and earlier studies retained where they remain the primary reference for a given mechanism or process. In essence, this review asks whether these emerging advances can be anticipated to hold for end-of-life vehicle foam, examining recovered-polyol quality, achievable blend ratios, the first industrial implementations, and the regulatory framework now generating demand before closing with the barriers that remain.

## 2. Polyurethane Foams in the Automotive Sector

**Figure 2:**
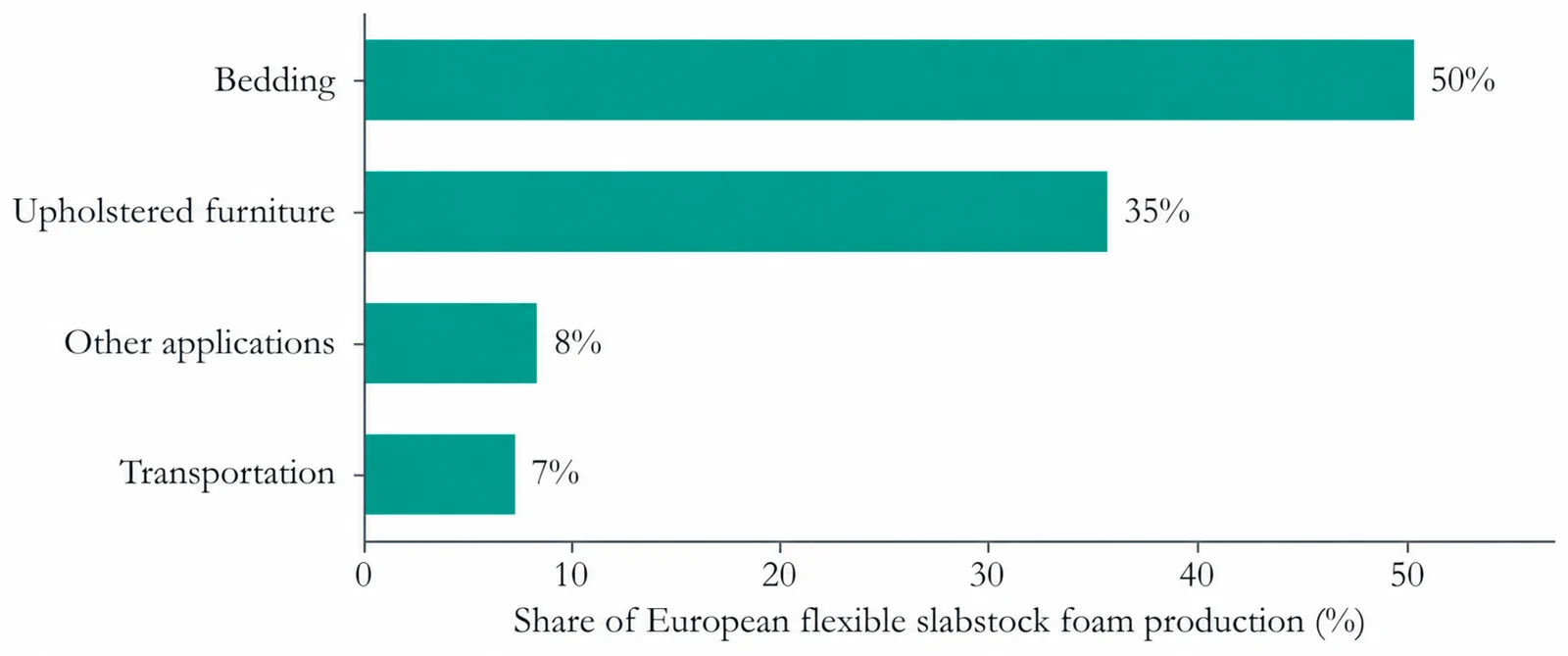
Applications of flexible polyurethane slabstock foam produced in Europe. Drawn using data from [22].

Polyurethane foam is the principal cushioning material in a vehicle interior. Flexible foam accounts for more than 90% of the padding used in car seats, and a typical passenger car carries on the order of 10–15 kg of it in the seats alone, with further flexible and semi-rigid foam in headrests, armrests, headliners, and acoustic packages [21]. The grade used in the cushions is a moulded foam of moderate density, firm enough to support an occupant through years of repeated loading yet light enough not to add unnecessary weight. The scale of this use is set out in Figure 2. Flexible foam accounts for roughly a third of European polyurethane consumption, and the slabstock portion alone accounts for approximately 1.5 million tonnes per year across some 160 factories in European Union, dominated by bedding and upholstered furniture, to which the moulded foam of car seats adds further volume [22]. Once these products reach end of life, the greater part is landfilled or incinerated rather than recycled [19].

**Figure 3:**
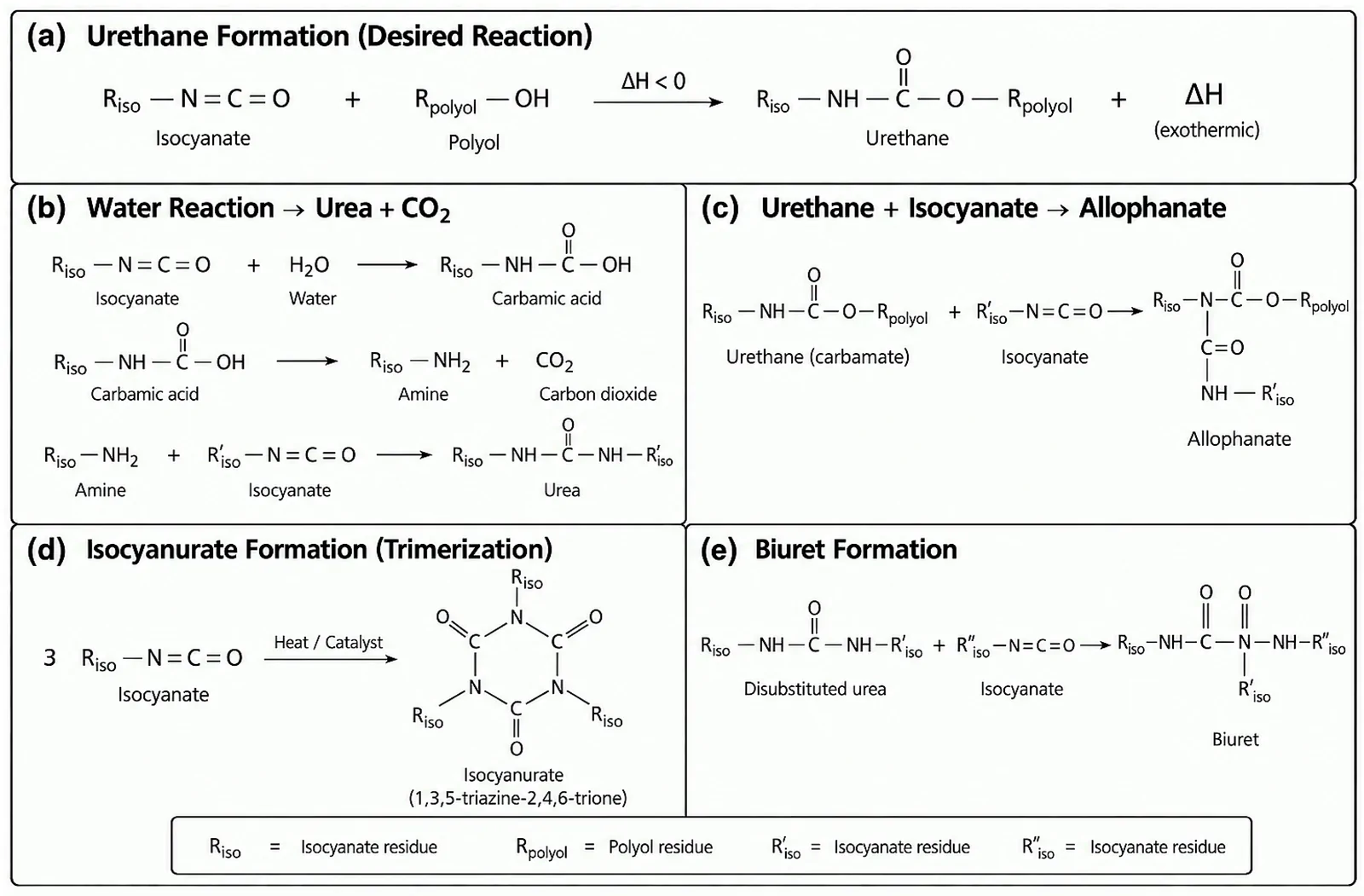
Polyurethane formation and its principal side reactions. (**a**) Isocyanate and polyol form the desired urethane linkage. (**b**) Water reacts with isocyanate to give CO_2_ and an unstable carbamic acid, which condenses with a further isocyanate to a disubstituted urea. (**c**) The carbamic-acid/urea intermediate reacts with a further isocyanate to give an allophanate branch point. (**d**) Isocyanate trimerises to an isocyanurate ring, releasing heat. (**e**) Disubstituted urea reacts with a further isocyanate to give a biuret branch point. (**a**,**b**) Adapted from [23]; (**c**,**e**) redrawn from [25]; (**d**) redrawn from [24].

The material forms when a polyol reacts with a di- or poly-isocyanate to create urethane linkages, the exothermic addition shown in Figure 3, together with the side reactions that the isocyanate group also undergoes (a–e) [23]. In a foam, water is commonly added as a blowing agent: it reacts with the isocyanate to give an unstable carbamic acid together with carbon dioxide, the gas that expands the mixture into a cellular network as it cures, and the carbamic acid reacts in turn with a further isocyanate group to form a disubstituted urea linkage (b). Because isocyanate is generally present in excess of what these two reactions consume, it also reacts further with the N–H of an already-formed urethane linkage, giving an allophanate branch point (c), or trimerises on itself, with three isocyanate groups condensing to a thermally stable isocyanurate ring with the release of heat (d), a reaction that underlies rigid polyisocyanurate foam and is promoted by dedicated trimerisation catalysts rather than occurring incidentally alongside the reactions above [24]. Disubstituted urea can react with a further isocyanate group in the same way, giving a biuret linkage (e); like the allophanate reaction, biuret formation is favoured by a higher isocyanate index and introduces a branch point beyond the crosslink density set by the polyol’s own functionality [25].

The two most common isocyanates are toluene diisocyanate (TDI) and methylene diphenyl diisocyanate (MDI), and varying these with the polyol and the degree of crosslinking shifts the product from soft, open-celled flexible foam to rigid, closed-celled insulation [26]. Within the flexible family, several grades are tailored to different roles in the vehicle, and these grades and their main automotive applications are summarised in Table 1.

**Figure 4:**
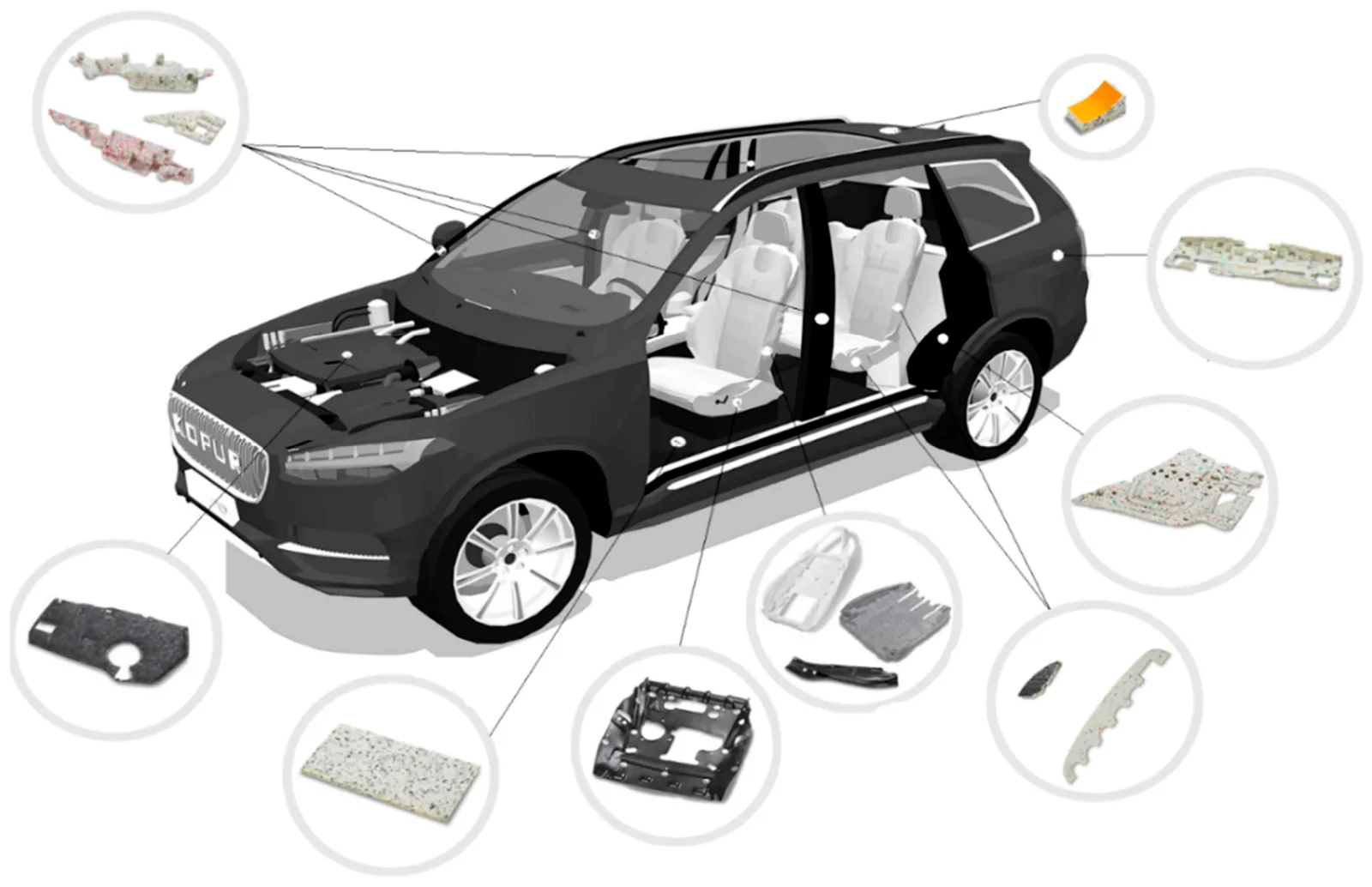
Distribution of polyurethane foam elements in a passenger car, from the acoustic fillers of the cabin to the moulded cushioning of the seats. Adapted from [29], licenced under CC BY 4.0.

Open-cell flexible foam became the standard seat-cushion material because it gives support comparable to the steel-spring assemblies it replaced at far lower weight and cost. Patten et al. reported that a good automotive seat must support the occupant in every driving posture, isolate the body from road vibration, and remain comfortable in temperature and humidity, stating that moulded flexible foam satisfies all of these. Vibration isolation is the most demanding requirement, since seated occupants are particularly sensitive to vertical motion near 6 Hz, referring to spinal resonance, so the cushion must damp motion around this frequency rather than amplify it [27]. The foam’s static and dynamic response is governed by the polymer’s molecular weight, its crosslink density, and the cell structure, which formulators adjust to balance comfort against durability [28]. The seat is only the largest of these applications: Figure 4 shows how foam elements are distributed through the vehicle, from acoustic fillers in the cabin to the moulded cushions of the seats [29].

Beyond the polyol and isocyanate, flexible foam is produced from a package of additives. According to the conventional two-component approach, surfactants, catalysts, and a blowing agent are combined on one side with the isocyanate on the other, each component serving to control the foaming reaction or to impart a required property to the finished part [30]. Among these, flame retardants are the most critical for automotive use, since untreated flexible foam is highly flammable, owing to its organic composition and open-celled structure; its limiting oxygen index has been reported to be only about 14 to 18% [31]. To satisfy vehicle fire-safety requirements, flame retardants are therefore incorporated as additives, introduced as reactive groups within the polymer, or applied as surface coatings, and these treatments have been shown to alter the thermal and mechanical behaviour of the foam [32]. Such additives are equally consequential at end of life, as they remain within the material and reduce the quality and purity of any recovered polyol, rendering ELV foam a more demanding feedstock than the clean industrial scrap commonly employed in recycling studies. Whether these additives survive solvolysis intact is a question the chemistry itself can answer, even where no study has yet tracked halogen or phosphorus balance through the glycolysis or acidolysis of automotive foam. Flexible foam is most often protected with the chlorinated phosphate ester triesters tris(1-chloro-2-propyl) phosphate, tris(1,3-dichloro-2-propyl) phosphate, tris(2-chloroethyl) phosphate, and the oligomeric analogue V6, together with non-halogenated aryl and alkyl phosphates and, less commonly, the brominated aromatic diesters that make up Firemaster 550 [33]. All of these are additive rather than reactive flame retardants, dispersed through the foam rather than bound into the polymer backbone, so once the cellular structure is broken down they partition freely into the glycol or diacid medium without any bond needing to be cleaved for their release. As a class, organophosphorus flame retardants are considerably more susceptible to hydrolytic and solvolytic attack at this ester linkage than their halogenated counterparts [34], so the chlorophosphates that dominate current flexible-foam formulations would be expected to undergo at least partial transesterification or hydrolysis under the elevated temperature and acid or base catalysis of glycolysis and acidolysis, releasing chlorinated alcohol fragments and phosphate or glycol-phosphate species into the crude product. Brominated aromatic esters are comparatively stable at these temperatures and decompose appreciably only above roughly 500 °C, well outside the range used in either route, so where they are present they more likely survive intact and simply partition between the polyol-rich and amine-rich phases [34]. A closely related solvolysis system offers a caution against treating either class as an inert contaminant: in the subcritical solvolysis of a flame-retarded carbon-fibre epoxy composite, phosphorus-, boron-, and metal-based additives neither degraded nor leached out but instead remained in the matrix and promoted a carbonised surface layer that hindered the treatment [35]. Which phase, the recovered polyol, the amine-rich fraction, or an off-gas, ultimately carries the degradation products of a flame-retarded ELV foam has not been established for any solvolysis route, and resolving it is a precondition for qualifying the recycled polyol against the flame-retardancy specifications the next foam must meet. Lowering the environmental footprint of automotive foam is not limited to recovering it at end of life, a parallel effort targets the virgin feedstock itself, and this context matters for what the recycling routes reviewed below will eventually act upon. A considerable body of research has investigated the partial replacement of petroleum-derived polyol with bio-based polyols obtained from plant oils, lignin, and other renewable sources, which can lower the carbon footprint of the foam without altering its fundamental chemistry [36]. Belsare and Nandi reported that a foam containing 10% bio-polyol satisfied the property requirements for automotive seating [37], while Dunne et al. demonstrated that a lignin-derived polyol could replace 20% of the fossil polyol while meeting the same requirements [38]. This substitution remains complementary to, rather than a substitute for, material recovery: the recycling routes addressed in the following section apply equally regardless of whether the original polyol was petroleum- or bio-derived.

## 3. Recovery Strategies for Polyurethane Foam

Recovering polyurethane foam has drawn increasing attention as priorities have shifted from disposal toward keeping the material in circulation, and several recycling routes have already moved beyond the laboratory into industrial-scale operation. Vehicle seats supply much of the flexible foam feeding these recovery streams, which places automotive foam at the centre of the recovery question [19]. Table 2 gathers the studies identified for this review that were conducted specifically on genuine automotive foam; assembling them in one place traces how the field’s attention to this particular feedstock has developed and points to where future work still needs to look. Even so, landfill and incineration remain the most common destination for waste foam, and the central task is to redirect it toward routes that preserve the polymer rather than break it down for its heating value alone.

The state in which the foam arrives complicates this task further. Post-consumer foam is seldom clean or uniform: it typically arrives mixed with other materials and contaminated, so any recovery route must cope with a variable feedstock rather than a single, well-defined polymer. Milling, the size-reduction step common to most routes, adds a further complication, since it releases the blowing agents held within the foam’s cells. In older foam, these agents included chlorofluorocarbons, which are liberated only slowly over many years and must be captured rather than vented [39]. Landfilling avoids these steps but offers no lasting resolution, since polyurethane resists natural biodegradation and is broken down only slowly and partially by a limited range of bacteria and enzymes [40,41].

**Table 2:** Chemical recycling studies conducted on genuine automotive polyurethane foam.

Author(s) and Year	Route	Automotive Feedstock	Ref.
Hicks, Kirk & Stapleton, 1996	Chemical recycling	MDI pour-in-place automotive seating	[42]
Beneš et al., 2007	Glycolysis	Flexible car-seat foam	[43]
Ko et al., 2023	Glycolysis + oxyalkylation	Post-consumer seat and headrest foam	[20]
Jašek et al., 2024	Glycolysis	Automotive headliner foam	[44]
Montag et al., 2026	Glycolysis	Automotive semi-rigid headliner foam	[45]
Conterosito et al., 2024	Glycolysis + purification comparison	Post-consumer car top padding	[46]
JLR/Dow/Adient, 2024	Glycolysis	Post-consumer seat foam	[47]

Foam recovery is accordingly divided into two conventional routes, mechanical and chemical, which differ in cost, simplicity, and the value of what they recover. These are examined in turn below.

### 3.1. Mechanical Recycling

**Figure 5:**
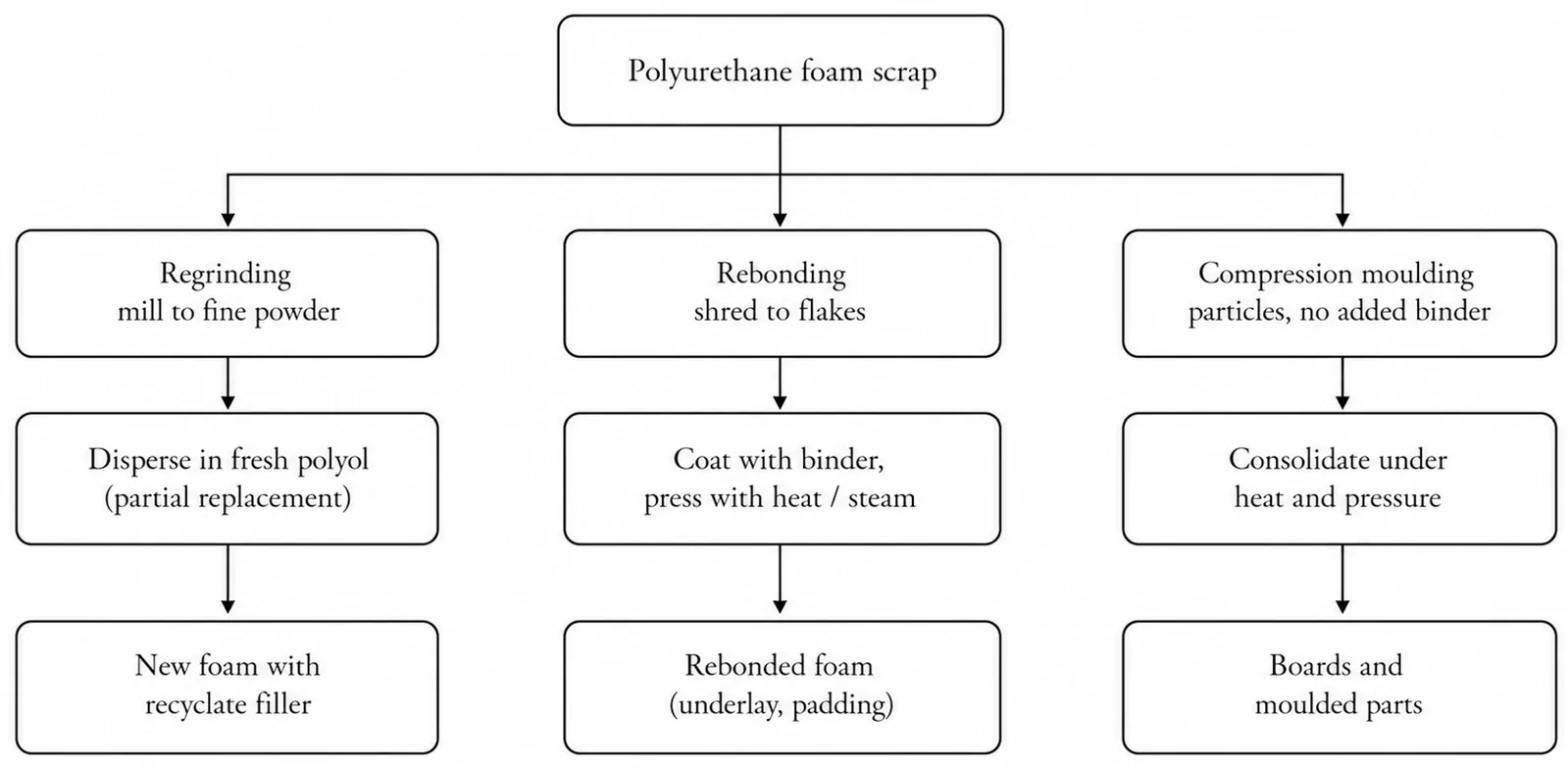
Mechanical recycling routes for polyurethane foam, grouped by working principle: regrinding to a powder dispersed in fresh polyol, rebonding shredded flakes with a binder under heat and steam, and consolidating particles under heat and pressure [26].

Mechanical recycling is the oldest and least expensive way of handling foam waste, and it remains the route that runs at the largest scale. Its principle is simple: the foam is broken down to a workable size and reformed into a new article without altering the chemistry of the polymer. Three approaches account for most of what is done: regrinding the foam to a powder, bonding shredded foam with an added binder, and consolidating foam particles under heat and pressure [26]. Each returns a product of lower grade than the polyol a chemical route yields, which marks the limit of the approach, yet each is cheap and quick and runs on equipment that recyclers already own [2]. Figure 5 groups these three approaches by their working principle.

**Figure 6:**
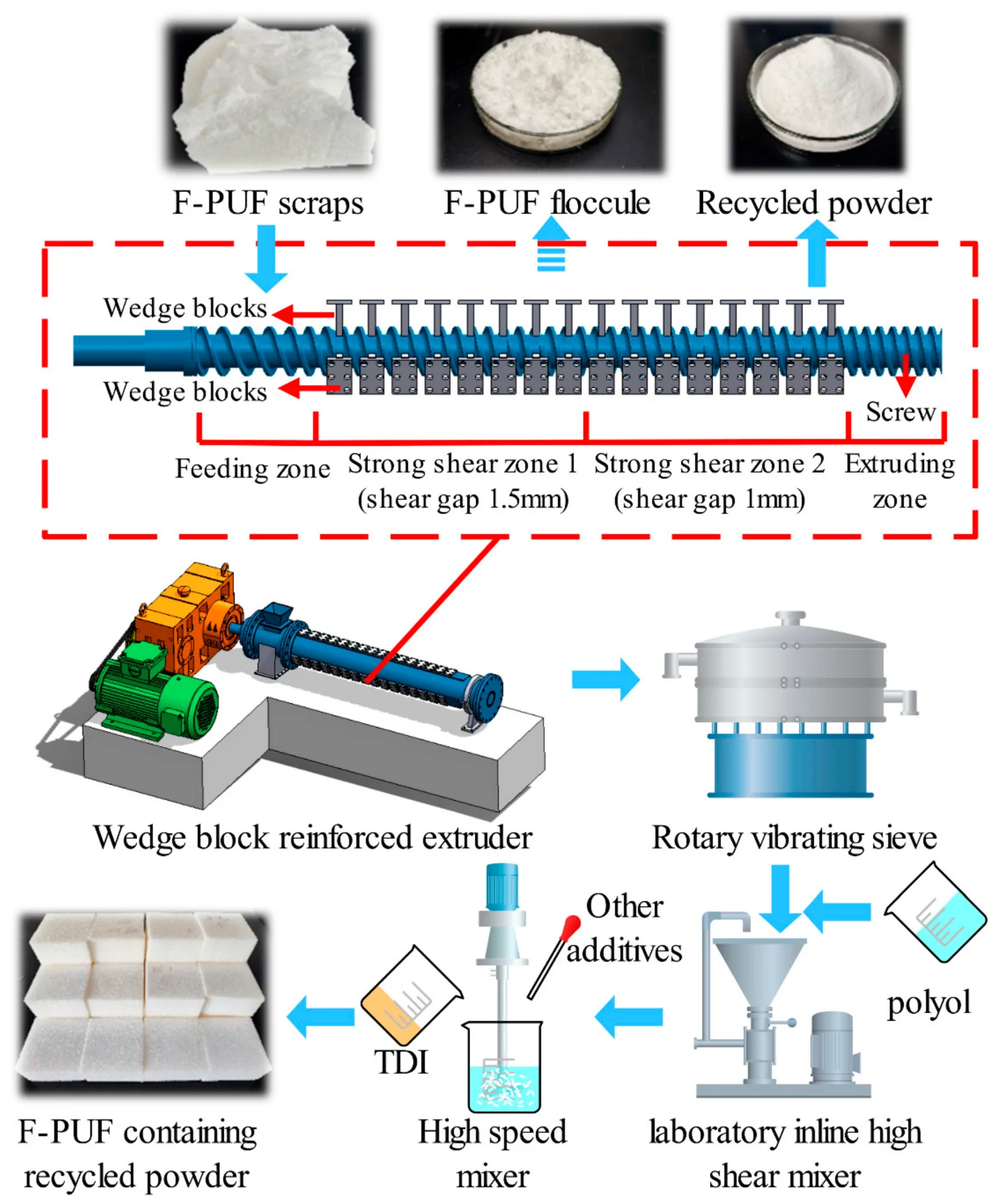
Mechanochemical recycling of flexible polyurethane foam in a wedge-block-reinforced extruder, in which repeated shear regenerates reactive surfaces for quantitative replacement of polyol. Adapted from [50], licenced under CC BY 4.0.

Regrinding is the most straightforward. Foam scrap is milled, often to a powder finer than a tenth of a millimetre, and the powder is stirred into the polyol component of a fresh formulation before the isocyanate is added so that it is carried into the regenerated foam as a filler [48]. The quantity taken back this way is modest because a heavy loading disturbs the foaming reaction and the cell structure it produces. Milling flexible foam on a two-roll mill shears and activates the particle surfaces, which lets the powder stand in for part of the virgin polyol rather than merely occupy space, and a powder near 100 µm prepared this way has replaced up to about 15 wt% of the polyol while keeping the resilience and compression set of the re-foamed product close to the original [49]. A reinforced extruder built on the same idea has since pushed towards near-complete replacement of the polyol [50]. Figure 6 illustrates this extrusion-based process, from foam scrap through the wedge-block screw to re-foaming with the recycled powder.

Rebonding works by glueing rather than dispersing. Foam is shredded to flakes about a centimetre across, coated with an isocyanate-terminated prepolymer that serves as the binder, and pressed in a heated mould at roughly 100 to 200 °C and pressures of up to about twenty megapascals until it sets to a chosen density, with steam passed through to complete the cure [51]. The bonded product is firmer than the scrap it came from and supplies roughly four-fifths of the carpet-underlay market, along with mats, packaging and acoustic padding [52]. Adhesive pressing follows the same binder-and-pressure principle while accepting a broader range of input, including the harder scrap left by reaction-injection moulding. Reground or rebonded foam is also combined with a thermoplastic and pressed into boards for thermal insulation [53].

The appeal of all of this, set against chemical recycling, is its cost. Mechanical recycling calls for no solvent or reagent, no vessel held for hours at temperature and no purification of a crude product, and life-cycle studies have repeatedly placed it as the lowest-impact of the recovery routes [19]. The economics point the same way. Recyclate can make up a large share of the input to hot compression moulding, which is what lets that route recover its cost while yielding parts that match the original [54]; a commercial-scale feasibility study of flexible-foam recycling found that returning ground foam to reconstituted foam at a replacement level near a tenth already trimmed costs by a few percent [55]. The trade is a plain one: mechanical recycling costs less and returns a lower-grade product, whereas chemical recycling costs more and can put the foam back to its first use.

Direct study of the mechanical recycling of foam taken specifically from car seats is limited, so the most useful evidence comes from the same family of flexible foam recovered from mattresses and furniture. Seat cushions are moulded flexible foam, usually built on MDI, while mattress foam is mostly TDI-based slabstock, but both are low-density flexible polyether foams that behave alike when shredded, ground or rebonded, which makes the mattress data a fair guide to the automotive case. An air-laid process that binds recovered mattress foam with a bi-component fibre produced a recycled layer soft enough to return to new mattresses, where rebonded foam alone would be too stiff [56], and recycled content in moulded automotive seating was demonstrated as far back as the 1990s [42]. The mattress and furniture stream is thus the working basis for judging what mechanical recovery of end-of-life vehicle foam can achieve, as well as the feedstock the rebonding industry already processes alongside automotive trim.

### 3.2. Chemical Recycling

Chemical recycling converts polyurethane foam back into a liquid raw material. The foam is heated with a reagent that cleaves its urethane and urea linkages, and from the resulting liquid the polyol can be separated, purified and returned to foam production. Simón et al. traced these processes from the first laboratory studies to industrial operation, where recovered polyols already replace part of the virgin polyol in new formulations [57]. Pillich et al. assessed the life-cycle impacts of chemically recycling rigid foam and concluded that a route is worth developing only where it performs better than the waste treatment already in place [58]. Four routes have been applied to polyurethane foam: glycolysis [59], acidolysis [60], hydrolysis [61] and aminolysis [62]. They differ in their reaction conditions and in the quality of the polyol they produce. Hydrolysis and aminolysis remain largely confined to laboratory investigations and are therefore noted here rather than treated in detail. Table 3 brings together the most recent advances in glycolysis and acidolysis, the two routes examined in detail in Section 3.2.1 and Section 3.2.2 that follow, so that the range of feedstocks and conditions reported can be seen at a glance. Setting these studies side by side also makes it possible to judge how well a route proven on another foam is likely to transfer to ELV foam and where that potential is still assumed rather than demonstrated.

Crosslink density is a further variable that the depolymerisation routes discussed above have to contend with, one this review has not yet addressed explicitly. Higher-functionality polyols, the building blocks that produce a more densely crosslinked network, are reported to give measurably longer dissolution times in glycolysis than the lower-functionality, longer-chain polyols used in flexible foam; rigid, highly crosslinked systems such as polyisocyanurate foam remain glycolysable only under more demanding conditions [59]. A more densely crosslinked network presents more urethane and urea junctions to cleave per unit volume and restricts diffusion of the depolymerising reagent through the network, a resistance to diffusion that increases with crosslink density in polymer networks generally [78], so both reaction rate and the depth of conversion reached in a given time are expected to fall as crosslink density rises. This matters directly for the foam grades summarised in Table 1: the moulded flexible and viscoelastic foam that fills seat cushions and headrests sits toward the lower end of this range, while the semi-rigid and acoustic grades used in instrument panels, door trim, and headliner and insulation backing are built at higher crosslink density to provide the stiffness or energy-absorbing structure those applications require. Glycolysis or acidolysis conditions validated on flexible seating foam should not, on this basis, be assumed to transfer directly to these more densely crosslinked grades, a possibility consistent with Jašek et al. [44] and Montag et al. [45] turning to specialised castor- and coconut-oil-derived reactants for headliner and semi-rigid material, in contrast to the diethylene or dipropylene glycol Beneš et al. [43] used on seat foam.

#### 3.2.1. Glycolysis

**Figure 7:**
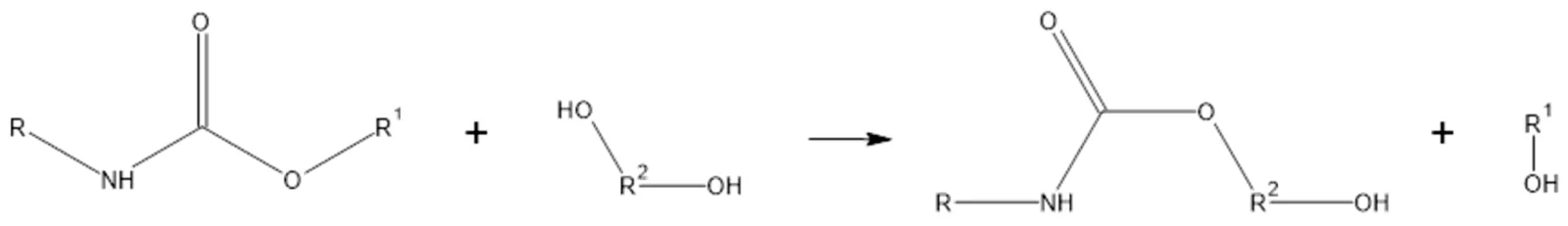
Glycolysis of the urethane linkage. Adapted from [59], licenced under CC BY 4.0.

**Figure 8:**
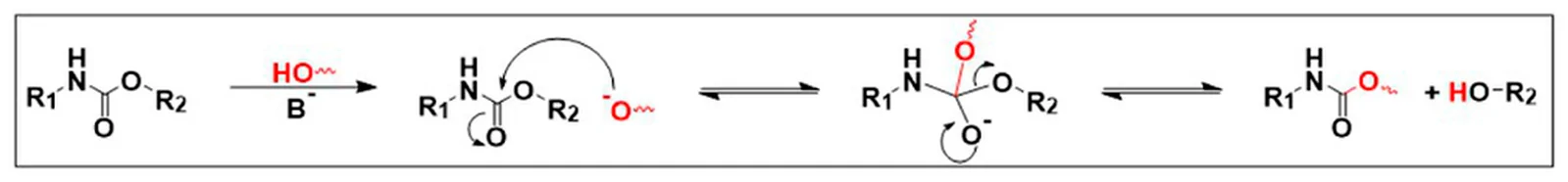
Base-catalysed transesterification mechanism of the urethane linkage. Adapted from [81] licenced under CC BY 4.0.

**Figure 9:**
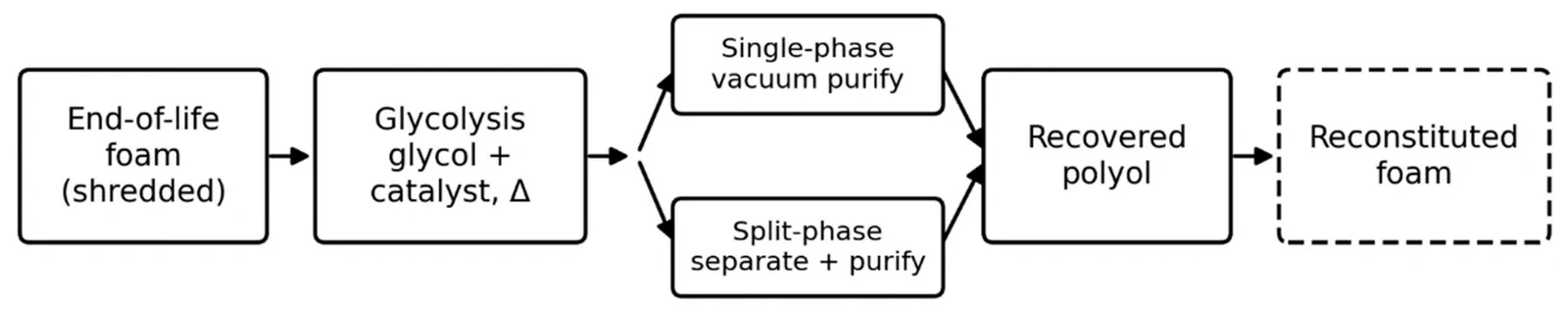
Process sequence for the glycolysis of polyurethane foam. Shredded foam is heated with a glycol and a catalyst, and the product is worked up either as a single phase or by separating and purifying the polyol layer of a split phase, giving a recovered polyol for return to reconstituted foam [20,79].

Glycolysis is the most developed of the chemical routes and the only one already running at industrial scale. A glycol, usually diethylene or dipropylene glycol, is heated with the foam and exchanges with the polyol at the urethane bond, releasing the polyether polyol while the nitrogen ends up in low-molecular-weight carbamate and urea fragments [18]. Figure 7 outlines this exchange, and Figure 8 follows the mechanism in more detail, showing the glycolate anion attacking the carbonyl carbon of the urethane group to form a tetrahedral intermediate that collapses to release the free polyol and form a new carbamate with the glycol. Once the reaction is complete, the product separates in one of two ways. In split-phase glycolysis, an upper polyol-rich layer forms above a denser, amine- and carbamate-rich glycol layer, driven by a polarity mismatch: the recovered polyol is comparatively non-polar, while residual glycol, carbamate, and urea fragments are strongly polar, so once their concentration exceeds what the polyol can hold in solution, the phases separate. Molero et al. (2006) showed that low-molecular-weight glycols such as diethylene glycol drive a clean split and give the highest-purity polyol, while bulkier glycols narrow the polarity gap and can prevent separation altogether [79]. In a follow-up study, Molero et al. (2008) found that a glycol-to-foam mass ratio near 1:1.5 maximises polyol purity (80–84 wt%) and minimises amine content, with higher ratios diluting the byproduct phase without further purity gain [80]. Single-phase processes avoid this separation step and reuse the whole glycolysate, trading purity for simplicity. Figure 9 summarises this process sequence, from shredded foam through either work-up route to the recovered polyol ready for return to reconstituted foam.

The route reached automotive foam early. Beneš et al. glycolysed flexible foam taken from car seats with diethylene or dipropylene glycol at 220 °C in 2007 and blended the recovered polyol into new rigid foam at up to 36 wt% without disturbing the process or the end-use properties [43]. Theirs was one of the few studies from its period to work with genuine seat foam rather than production scrap, and it established that car-seat polyol could be recovered and reused without special handling.

More than a decade passed before the next demonstrations followed, and these deserve the closest attention because they work with the feedstock this review is concerned with. Ko et al. noted that industrial glycolysis had concentrated almost entirely on mattresses, with automotive foam having been rarely attempted because of its comfort, durability, and hygiene specifications. In 2023, they presented the first efficient single-phase glycolysis of post-consumer automotive foam, coupling it with an oxyalkylation step to stabilise the recovered polyol. The product was re-foamed into seats, headrests, and sound-absorbing parts at 10–20 wt% [20]. Jašek et al. carried automotive headliner foam through glycolysis in 2024 with 2-hydroxypropyl ricinoleate, a castor-oil-derived reactant, and ran the process at 125 L; the recovered polyol raised the tensile and flexural strength of the re-formulated foam rather than weakening it, pointing to a recyclate good enough for demanding automotive parts [44]. Montag et al. then closed the loop on automotive semi-rigid headliner foam in 2026 using a coconut oil-based reactant, recovering polyol and returning it to a foam of the same type [45]. Together, these four studies cover seat, headrest, and headliner foam, extend from pilot to industrial scale, and report recovered polyols that meet automotive requirements; they are the most direct demonstrations to date showing that glycolysis is transferable to genuine vehicle foam.

Set against this automotive-specific record, most glycolysis work has instead been carried out on mattress, slabstock, and rigid feedstocks, chemically distinct from the MDI-based moulded foam of a car seat or the semi-rigid grades used in headliners yet built on the same urethane chemistry. Kiss et al. compared atmospheric, autoclave, and microwave heating on flexible ester foam and reused the whole glycolysate without separation [63]. Donadini et al. tackled the free aromatic amines that lower polyol quality, deaminating the glycolysis product directly [82], while Peng et al. removed the separation step altogether with a single-phase route [83]. Greener reactants have also been tried on these feedstocks: Chang et al. and Fu et al. both replaced conventional glycols with crude glycerol, a biodiesel by-product, recovering single-phase biopolyols from rigid and refrigerator foam and, in the second case, splitting the product into an oil–water separation sponge and a rigid insulating foam [64,65]. Velasco et al. demonstrated a scalable ionic-liquid-assisted glycolysis [66], and Pang et al. reported a solvent-assisted process giving recovered polyol reusable without purification at up to 40 wt% in rigid foam, with a global-warming potential about 40% below the virgin route [67]. Jung combined waste polyurethane and poly(ethylene terephthalate) in a single glycolysis-and-aminolysis step to make a flame-retardant insulation foam [84], and Johansen et al. used a tert-amyl alcohol solvolysis that recovers both the polyol and the aniline fraction [85]. None of these studies used automotive foam, yet the reaction relies on glycol exchange at the urethane bond and is the same reaction that Beneš, Ko, Jašek, and Montag ran on genuine seat, headrest, and headliner material. Because the underlying chemistry does not distinguish between a mattress slab and a moulded seat cushion, a technique validated on one feedstock has a reasonable claim to transfer to the other, and the gains reported on mattress and rigid foam—cleaner separation, lower amine content, greener reactants—are exactly the improvements ELV foam most needs. This is the strongest argument for directing more of this work toward automotive material specifically: the chemistry has already been shown to travel between feedstocks, so what is missing is not proof of principle but validation on the contaminated, multi-component foam a scrapped vehicle actually yields. One aspect of that validation gap is worth flagging specifically: how much contamination, fabric, adhesive residue, and flame retardants a glycolysis process can absorb before it stops being economically worthwhile. No study has established this threshold, nor compared single-phase against split-phase tolerance. The closest evidence is indirect: Conterosito et al. found that purifying automotive-derived glycolysate from 4.26% to 1.32% residual nitrogen raised the workable polyol substitution from 15 to 30 wt% [46]. On this basis, and reasoning from each process’s mechanism, single-phase glycolysis retains the whole glycolysate, is likely limited to weight percent of particulate contamination before the cell structure suffers, while split-phase separates contaminants into the amine-rich phase and can probably tolerate more, perhaps 5–10 wt%, until contamination instead prevents clean phase separation altogether. These are reasoned estimates, not established values, and testing them on real ELV feedstock remains a priority for future work.

A separate line of work focuses on designing foam to be recycled in the first place. Gotkiewicz et al. built bio-based ester-cleavable segments into semi-rigid foam that glycolyses quickly, returning up to 50 wt% recovered polyol to new rigid foam, and developed an ultralow-density variant on the same principle [68,86]. Kassem et al. integrated β-amino-ester units that allow a foam-to-foam loop, with full disintegration in twenty minutes at 65 °C [69], and Kurańska et al. recovered reusable rebiopolyols from bio-based foam [87]. These results are promising, but they rely on purpose-built chemistries rather than the conventional foam that fills a vehicle today, so their relevance to the existing ELV stream is indirect. Taken as a whole, glycolysis is both the most mature route and the one with the clearest automotive demonstrations.

#### 3.2.2. Acidolysis

**Figure 10:**
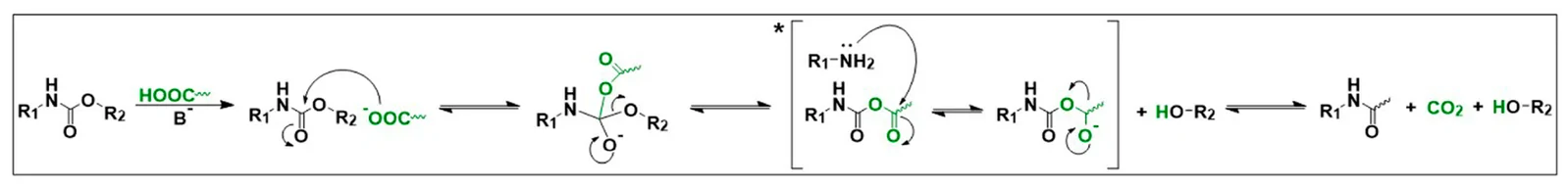
Mechanism of acidolysis at the urethane and urea linkages. Asterisk (*) indicates the urea-specific reaction pathways involving the amine intermediate. Adapted from [81], licenced under CC BY 4.0.

Acidolysis is the newer route, and it tends to give cleaner polyols under milder conditions than glycolysis. A dicarboxylic acid cleaves both the urethane and the urea bonds, releasing the polyol while converting the nitrogen-bearing fragments into amines and amides. Gama et al. established the approach using polyurethane scraps in 2020, determining how reaction conditions set the hydroxyl number, acid value, and viscosity of the recovered polyol [71]. Godinho et al. then extended it across polyester, polyether, and viscoelastic foam and carried the recovered polyol forward into wood coatings, showing that the route is not tied to a single foam chemistry or a single end product [70]. Figure 10 sets out the mechanism behind both steps.

**Figure 11:**
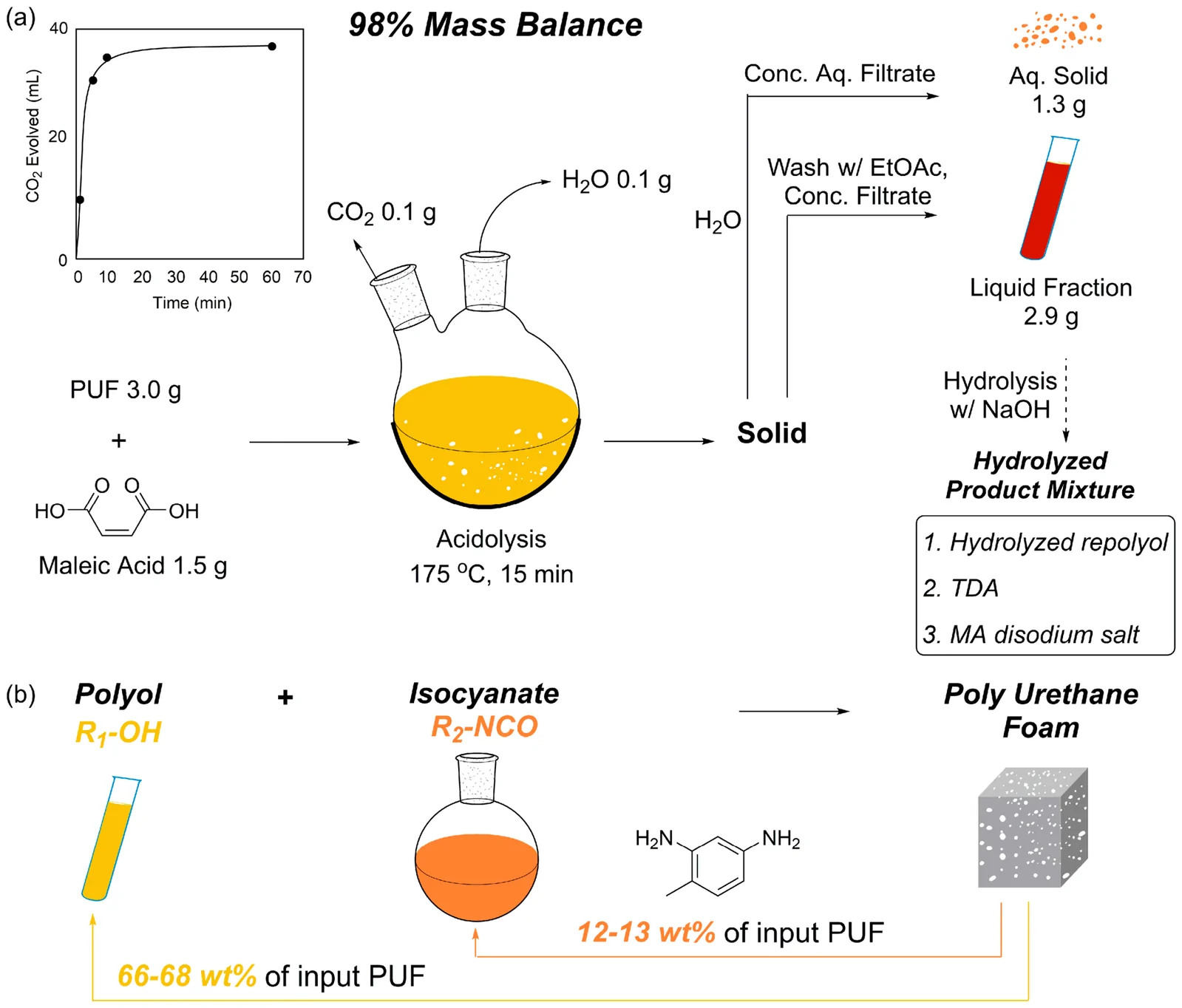
Acidolysis of flexible polyurethane foam with maleic acid (**a**) and return of the recovered products to foam production (**b**). Fifteen minutes at 175 °C gives a 98% mass balance, with the repolyol and the toluenediamine both re-entering the foam loop. Adapted from [91], licenced under CC BY 4.0.

The mechanism matters for ELV foam because it predicts how clean the recovered polyol will be. The diacid attacks both linkages: cleaving the urethane releases the polyether polyol, the product of interest, while cleaving the urea frees the aromatic amine and leaves an acid-functionalised amide, which closes into an imide when the diacid is short-chained [88]. Westman et al. mapped how the structure of the acid governs the kinetics across roughly ten dicarboxylic acids, describing polyol release with a shrinking-core model [77], and showed that the same structure controls the distribution of nitrogen-containing products [89]. Davis et al. separated the two bonds outright, finding biphasic behaviour in which the urethane cleaves quickly and the urea more slowly, with the urea rate sensitive to the electronic character of the acid [90]. The practical message is that the residual amine content of the recovered polyol, which is what most limits its reuse, can be tuned through the choice of acid. Figure 11 shows these products emerging in practice: in maleic-acid acidolysis, carbon dioxide evolves within the first minutes as the linkages are consumed, and the foam resolves into the three fractions the mechanism predicts, the recovered polyol from the urethane, the aromatic diamine freed from the urea, and the spent acid, with the mass balance closing at 98% [91].

**Figure 12:**
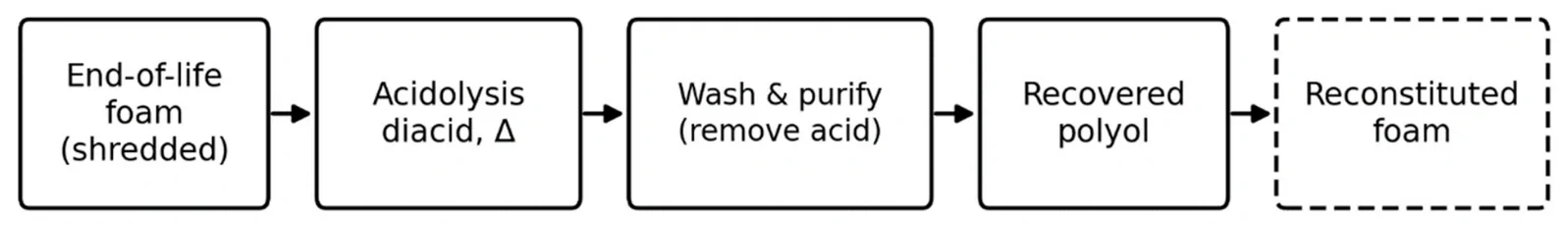
Process sequence for the acidolysis of polyurethane foam. Shredded foam is heated with a dicarboxylic acid under milder conditions than glycolysis, and the product is washed and purified to remove residual acid, giving a recovered polyol for return to regenerated foam [71,88].

On the process side, several groups have advanced the route toward cleaner polyols and shorter processing times. Grdadolnik et al. used adipic acid under microwave heating and cleaved most of the urethane within fifteen minutes [72]. He et al. ran a solvent-free acidolysis catalysed by zinc acetate, reaching 97% degradation in three hours and substituting up to 40 wt% of virgin polyol in new flexible foam [73]. Liu et al. recovered polyol fully and quickly with maleic acid [74], and Aksu et al. followed a maleic-and-phthalic recyclate into flexible foam across a 10–50 wt% range, finding that the properties held to about 20 wt% before resilience and air permeability fell away [75]. Omrani and Mohammadi Berenjegani moved toward greener inputs with bio-based acidolysis agents [76]. Compared with glycolysis, these studies report milder temperatures and cleaner polyols, though acid value and residual amine remain the properties to watch. Figure 12 outlines the process sequence, from shredded foam through washing and purification to the recovered polyol.

Acidolysis is advancing quickly and rests on a solid mechanistic foundation, yet none of this work has been demonstrated on genuine automotive foam: the feedstocks studied so far have been flexible slabstock, mixed scrap, or foam prepared in the laboratory, in contrast to the glycolysis studies of Ko, Jašek, and Montag. Whether the route can be carried across to genuine car-seat or headrest material, as glycolysis has been, is examined in the comparative assessment that follows.

## 4. Comparative Assessment of Chemical Recycling Routes for Elv Foam

Glycolysis and acidolysis both recover polyol from polyurethane foam, yet their standing with respect to the end-of-life vehicle stream differs markedly. Glycolysis is the more mature of the two: it operates at industrial scale, and its automotive credentials rest on genuine seat, headrest, and headliner foam, demonstrated respectively by Ko et al., Jašek et al., and Montag et al. [20,44,45]. Acidolysis, by contrast, has not yet been tested on any automotive feedstock; the studies discussed above worked exclusively with slabstock, mixed scrap, or laboratory-prepared foam. This asymmetry, however, reflects the extent of testing rather than a difference in the underlying chemistry. Both routes cleave the same urethane and, where applicable, urea linkages; the transfer that glycolysis has already achieved—from mattress and industrial feedstocks to genuine vehicle foam—gives no particular reason to expect acidolysis to behave otherwise once it receives comparable scrutiny. Table 4 sets the four depolymerisation routes against one another on criteria that determine industrial suitability: technology readiness, industrial implementation, applicability to automotive feedstocks, recovered-polyol quality, and process-related limitations.

Glycolysis’s industrial standing is not without cost. Established process know-how and commercially available equipment have secured its integration into existing polyurethane supply chains, yet the aromatic amine by-products it generates and the purification burden they impose remain a persistent economic and environmental liability [57]. Acidolysis is attractive precisely where glycolysis is weakest: its lower reaction temperatures and cleaner polyol output address the purification problem directly [71]. What it lacks is scale, not principle. Its technology readiness trails glycolysis, no automotive trial has yet been reported, and scale-up experience remains limited, so a direct industrial comparison between the two routes is premature rather than unfavourable to acidolysis.

Hydrolysis and aminolysis remain confined to laboratory-scale investigation, and neither has generated evidence of applicability to automotive polyurethane foam. On the balance of evidence assembled here, glycolysis warrants adoption as the reference technology for near-term ELV recycling, whereas acidolysis stands as the most promising candidate for the technology that follows it, contingent on validation against genuine, contaminated, ELV-derived feedstock rather than the cleaner material on which it has so far been proven.

## 5. Current Challenges and Prospects

The barriers that separate this chemistry from routine use lie mostly outside the reactor, in process integration and industrial deployment rather than in the reaction itself. The first barrier concerns the feedstock itself. The post-industrial headliner scrap processed by Montag et al. was clean and mono-component, whereas a cushion recovered from a scrapped car carries fabric facings, flame retardants, soiling, and adhesive [45]. Martinez Sanz et al. identified poor separation and sorting as a principal weakness of end-of-life vehicle plastics recovery in general, since components not removed during dismantling were reported to become a shredder residue too heterogeneous for material recovery, and the dismantling that would keep the foam separate competes against cost with shredding the complete hulk [2]. The second barrier concerns the recovered polyol. Solvolysis of an aromatic-isocyanate foam liberates aromatic amines, chiefly 4,4′-methylenedianiline, a listed substance of very high concern, and although their removal has been demonstrated for glycolysis products, the additional purification adds cost to a process whose margin is already narrow [82]. Several strategies have been reported for keeping 4,4′-methylenedianiline and other aromatic amines out of the recovered polyol. Chemical scavenging treats the crude glycolysate directly with a reagent that consumes the free amine: Donadini et al. found that an epoxide, 2-ethylhexyl glycidyl ether, cut the 4,4′-methylenedianiline content of a model polyol from about 7000 to 100 ppm at 99% conversion, outperforming an acylating agent that left more residual amine and weakened the resulting foam [82]. In situ conversion instead recovers the amine as a usable co-product during depolymerisation itself, as in Johansen et al.’s solvolysis, which isolates the polyol and aniline fraction separately [85], and Van Belleghem et al.’s analogous ammonolysis of rigid foam [62]. A third route relies on post-reaction extraction, such as the solvent-extraction separation of methylenedianiline, isophoronediamine, and toluenediamine reported by Eberz et al. [92]. None of these has yet been demonstrated on genuine ELV foam. A third barrier is composite construction: a headliner is a sandwich of semi-rigid foam between glass-fibre mats beneath a textile cover, and recovering the foam requires the separation of materials that were bonded to act as one.

Qualification presents an additional barrier: regardless of the production cost of a recovered polyol, it must meet the specifications required for automotive components. Wieczorek et al. incorporated a glycolysate recovered from semi-rigid construction foam into rigid insulation foam at loadings up to half the polyol fraction and characterised the product for foaming kinetics, thermal conductivity, dimensional stability, flammability, and volatile-organic-compound emissions. Recycled content shortened the gel time and permitted a reduction in catalyst loading, while emissions and dimensional behaviour set the practical ceiling on the recyclate content that a performance-rated foam can carry [93]. These are the same properties against which an interior automotive foam is qualified, and this work, rather than the depolymerisation chemistry, is likely to set the pace at which recovered polyol reaches production components.

### Emerging Developments and Research Directions

The first industrial implementations have been reported, and they are based on glycolysis. In 2024, Jaguar Land Rover, Dow, and Adient reported the first closed-loop reuse of seat foam recovered from end-of-life vehicles: the foam was collected, shredded, and depolymerised to a recovered polyol that supplied 20% of the foam in prototype seat cushions, with production-scale testing to follow [47]. Specifications for properties such as reaction temperature, pressure, catalyst, the glycol used, and the mechanical performance of the resulting foam are not available; being an industry press release rather than a peer-reviewed technical report, the announcement was not intended to provide this level of detail, and the case illustrates how commercially sensitive process information can limit independent scrutiny of early industrial implementations. Whether such processes operate at the scale and cost that a vehicle programme requires remains an open question for the wider industry, and Del Amo et al. addressed it for glycolysis. Applying geometric and dynamic similarity to scale a split-phase process from laboratory results to pilot plant, they costed a facility treating 270 tonnes of flexible foam scrap per year and reported a net present value near 1.46 million euro, an internal rate of return close to 28%, and a payback period of four to five years, with crude glycerol, a by-product of biodiesel manufacture, as the cleavage agent [94]. Favourable economics at modest scale matter for a feedstock that reaches dismantlers in dispersed and variable quantities, and the trajectory these demonstrations define, from pilot-scale economics to qualification against automotive specifications, is the one a successor route such as acidolysis would follow. The research that would consolidate this progress separates into three directions.

The first concerns environmental accounting. The 2025 eco-profile of European flexible polyurethane foam, an ISO-compliant cradle-to-gate assessment of the industry’s production, attributes more than 85% of the impact, in all but two of the categories analysed, to its precursors, the polyether polyol and the isocyanate; for foam grades without flame retardant, the flame-retardant grade follows the same pattern but with a lower precursor share [22]. Recovering the polyol therefore addresses the largest source of the foam’s environmental burden, since every kilogram of recovered polyol displaces the most impact-intensive input of the next foam. What remains scarce is route-specific assessment on genuine automotive feedstock; supplying it would show manufacturers which of the processes reviewed here could repay scaling. Such assessments should evaluate greenhouse gas emissions, cumulative energy demand, solvent and reagent consumption, and the impacts of purification, rather than material recovery rates alone.

The second direction concerns the material itself. The foams now reaching end of life were not designed for disassembly, and the more durable remedy is to design the next generation so that they are designed for disassembly. Miravalle et al. reviewed covalent adaptable networks in polyurethanes, in which dynamic bonds allow a crosslinked foam to be reprocessed under heat in a way a conventional thermoset resists [95]. This promise comes with a processing window that is considerably narrower for a flexible cushioning foam than for a solid moulding. Dynamic exchange, whether transesterification, disulfide exchange, or the carbamate exchange native to polyurethane itself needs a catalyst at effective concentration and enough time and temperature to let the network flow, and flexible foam is made and used under conditions that work against both. Sheppard et al. reprocessed postconsumer foam, including a flexible open-cell grade, by soaking the ground material in a dibutyltin dilaurate solution to introduce a bond-exchange catalyst that the original formulation lacked, extruding it at 200 °C; even so, the roughly one-minute residence time barely relaxed the stress, leaving an extrudate resistant to creep but needing a fresh catalyst for every further cycle [96]. The same bond mobility that lets a network reprocess at elevated temperature also lets it relax at whatever temperature it sees in service, and a car-seat cushion has to resist exactly that, creep and permanent set under years of sitting load, in a cabin that can exceed 80 °C in direct sun. The formulation task this sets up is therefore more specific than “make the foam reprocessable”: the bond-exchange catalyst needs to stay effectively dormant across the foam’s normal service-temperature range and activate only under the deliberately harsher conditions of a dedicated reprocessing step; building that dormancy in from the start, rather than soaking it in afterward as Sheppard et al. [96] did, is what would let a CAN foam actually reach the seat without trading away its dimensional stability. Delavarde et al. surveyed the broader movement toward sustainable polyurethanes, including bio-based polyols, isocyanate-free chemistries, and greener processing, each of which alters what an end-of-life foam will consist of in a decade [97]. Recoverability designed into the material reduces the demands placed on the recovery step.

The third direction concerns the recovery process. The reagents and conditions in current use descend from decades of glycolysis practice, and milder alternatives are emerging. Salas et al. depolymerised foam waste hydrolytically with ionic liquids under conditions gentler than conventional glycolysis [98]. Biological routes are approaching feasibility: Yu et al. reviewed the microbial and enzymatic depolymerisation of polyurethane together with the valorisation of the resulting monomers, and enzymatic cleavage proceeds at ambient temperature with a selectivity suited to the mixed and contaminated foam yielded by a scrapped vehicle, although reported rates remain below industrial throughput [99].

Consequently, future research should focus not only on product quality and depolymerisation efficiency but also on scale-up feasibility, process robustness, and integration into existing automotive recycling infrastructures.

## 6. Conclusions

Polyurethane foam has been treated as an afterthought in end-of-life vehicle recycling, and the evidence gathered here suggests that view no longer holds. Industry already runs glycolysis at industrial scale to recover it; what remains untested is whether the same result holds on the foam a scrapped car actually produces, rather than on the cleaner mattress and industrial scrap used to prove the chemistry. Closing that gap, not finding new chemistry, will decide whether polyurethane foam becomes part of the vehicle industry’s recycled content going forward. Four things would move that gap closer to closing include the following: testing both established and emerging routes directly on foam collected from dismantling operations rather than cleaner substitutes, working out how to strip aromatic amines from the recovered polyol without adding prohibitive cost, running the techno-economic and life-cycle assessments this feedstock still lacks, and qualifying the resulting polyol against the performance specifications a vehicle interior demands. Whether polyurethane foam ends up contributing meaningfully to the recycled content that the forthcoming European ELV Regulation will require depends on how quickly this work gets done.

## Figures and Tables

**Table 1:** Flexible polyurethane foam types and their main automotive applications.

Foam Type	Cell Structure	Key Property	Main Automotive Uses
Moulded flexible (high-resilience)	Open-cell	Resilient, load-bearing comfort	Seat cushions, seat backs, headrests, armrests
Viscoelastic (memory)	Open-cell	Slow recovery, pressure distribution	Comfort layers in premium seating
Semi-rigid	Mostly open-cell	Energy absorption stiffness	Instrument panels, door trim, knee bolsters, crash padding
Acoustic	Open-cell reticulated	Sound absorption and damping	Headliner backing, dash and floor insulation, carpet underlay

**Table 3:** Summary of recent chemical recycling studies on polyurethane foam, grouped by depolymerisation route.

Author(s) and Year	Route	Foam Feedstock	Reagent/Conditions	Ref.
Kiss et al. 2020	Glycolysis	Flexible ester foam waste (industrial)	Diethylene glycol	[63]
Chang et al. 2025	Glycolysis	Rigid refrigerator foam	Crude glycerol	[64]
Fu et al. 2025	Glycolysis	Flexible foam waste	Crude glycerol	[65]
Velasco et al. 2025	Glycolysis	Flexible foam waste	Ionic-liquid catalyst	[66]
Pang et al. 2026	Glycolysis (solvent-assisted)	Flexible foam waste	Polyether polyol with small-molecule diols	[67]
Gotkiewicz et al. 2025	Glycolysis	Semi-rigid foam with bio-based ester	Built-in ester segments; microwave	[68]
Kassem et al. 2024	Glycolysis	Flexible foam with β-amino-ester units	Mild glycolysis	[69]
Godinho et al. 2021	Acidolysis	Polyester, polyether	Dicarboxylic acids	[70]
Gama et al. 2020	Acidolysis	Flexible PU foam scraps	Succinic acid	[71]
Grdadolnik et al. 2022	Acidolysis	Flexible foam	Adipic acid, microwave heating	[72]
He et al. 2023	Acidolysis	Flexible foam	Zinc acetate catalyst, solvent-free	[73]
Liu et al. 2024	Acidolysis	Flexible foam	Maleic acid	[74]
Aksu et al. 2025	Acidolysis	Flexible foam	Maleic and phthalic acid	[75]
Omrani and Mohammadi Berenjegani 2024	Acidolysis	Flexible foam	Bio-based acidolysis agents	[76]
Westman et al. 2024	Acidolysis	Model and end-of-life flexible foam	About ten dicarboxylic acids (kinetic study)	[77]

**Table 4:** Critical comparison of polyurethane recycling routes for ELV applications.

Parameter	Glycolysis	Acidolysis	Hydrolysis	Aminolysis
Technology readiness	High	Medium	Low	Low
Industrial implementation	Yes	No	No	No
Automotive validation	Yes	No	No	No
Polyol quality	Medium–High	High	High	High
Aromatic amine formation	Significant	Moderate	Low	Moderate
Typical operating temperature	High	Moderate	High	Moderate
Purification requirements	High	Moderate	Moderate	Moderate
Applicability to ELV foam	Demonstrated	Expected	Unknown	Unknown

## Data Availability

No new data were created or analyzed in this study. Data sharing is not applicable to this article.
